# Helicase-like transcription factor expression is associated with a poor prognosis in Non-Small-Cell Lung Cancer (NSCLC)

**DOI:** 10.1186/s12885-018-4215-y

**Published:** 2018-04-16

**Authors:** Ludovic Dhont, Melania Pintilie, Ethan Kaufman, Roya Navab, Shirley Tam, Arsène Burny, Frances Shepherd, Alexandra Belayew, Ming-Sound Tsao, Céline Mascaux

**Affiliations:** 1 Laboratory of Molecular Biology, Research Institute for Health Sciences and Technology, Université de Mons, Mons, Belgium; 2 Princess Margaret Research Institute, Princess Margaret Cancer Centre, University Health Network, Toronto, Canada; 3 Cellular and Molecular Epigenetics, Université de Liège-GIGA, Liège, Belgium; 4 Biostatistics Department, University of Toronto, Toronto, Canada; 5 Université Libre de Bruxelles (ULB), Bruxelles, Belgium; 6 Division of Medical Oncology and Hematology, Princess Margaret Cancer Centre, University Health Network, Toronto, Canada; 7 Laboratory of Medicine and Pathobiology, University of Toronto, Toronto, Canada; 8 Department of Muldisciplinary Oncology and Therapeutic Innovations, Assistance Publique des Hôpitaux de Marseille (AP-HM), Aix-Marseille University, Chemin des Bourrely, 13195 Marseille, Cedex 20, France; 9 Centre de Recherche en Cancérologie de Marseille (CRCM, Cancer Research Center of Marseille), Inserm UMR1068, CNRS UMR7258 and Aix-Marseille University UM105, Marseille, France

**Keywords:** Non-small cell lung cancer, HLTF, Prognosis, Alternative splicing

## Abstract

**Background:**

The relapse rate in early stage non-small cell lung cancer (NSCLC) after surgical resection is high. Prognostic biomarkers may help identify patients who may benefit from additional therapy. The Helicase-like Transcription Factor (HLTF) is a tumor suppressor, altered in cancer either by gene hypermethylation or mRNA alternative splicing. This study assessed the expression and the clinical relevance of wild-type (WT) and variant forms of *HLTF* RNAs in NSCLC.

**Methods:**

We analyzed online databases (TCGA, COSMIC) for *HLTF* alterations in NSCLC and assessed WT and spliced *HLTF* mRNAs expression by RT-ddPCR in 39 lung cancer cell lines and 171 patients with resected stage I-II NSCLC.

**Results:**

In silico analyses identified *HLTF* gene alterations more frequently in lung squamous cell carcinoma than in adenocarcinoma. In cell lines and in patients, WT and I21R *HLTF* mRNAs were detected, but the latter at lower level. The subgroup of 25 patients presenting a combined low WT *HLTF* expression and a high I21R *HLTF* expression had a significantly worse disease-free survival than the other 146 patients in univariate (HR 1.96, CI 1.17–3.30; *p* = 0.011) and multivariate analyses (HR 1.98, CI 1.15–3.40; *p* = 0.014).

**Conclusion:**

A low WT *HLTF* expression with a high I21R *HLTF* expression is associated with a poor DFS.

## Background

Lung cancer is responsible for the highest cancer-associated mortality rate worldwide. Only 16% of patients affected with Non-small cell lung cancer (NSCLC), which is the most common subtype, are alive 5 years after diagnosis, and this number has hardly improved over several decades [1]. One reason for this poor prognosis is that only 15% of lung cancers are diagnosed at an early stage. Till recently the standard of care for NSCLC at stages I-IIIA was surgery, resulting in patient survival rates of 23% in stage IIIA, 33% in stage IIB, and up to 89% in stages IA [2]. Adjuvant chemotherapy after radical resection of localized NSCLC improves survival at 5 years by about 5% [3]. However, there is still a relatively high risk of relapse, and up to 40% of all stage IB and 60% of stage II patients die from their disease despite receiving adjuvant chemotherapy [4]. The integration of prognostic and predictive biomarkers has the potential of identifying patients who are at a low-risk of relapse following surgery and do not need further therapy, and conversely, patients who are at a high risk of relapse and who potentially may derive the greatest benefit from adjuvant treatment, including chemotherapy or personalized treatment based on individual tumor profiling. Therefore, an effort to identify more robust prognostic and predictive biomarkers is needed [5].

The Helicase-like Transcription Factor (HLTF) is a member of the yeast mating SWItch/Sucrose Non Fermenting (SWI/SNF) family of proteins involved in chromatin remodeling. Several studies demonstrated its function in gene transcription [6], cell cycle [7], DNA repair [8, 9], and genome stability maintenance [10], supporting its tumor suppressor role. In cancer, two different alterations in *HLTF* expression were reported: (i) an epigenetic silencing by hypermethylation of its promoter and (ii) an alternative splicing of its mRNA, leading to the production of several shorter forms of the protein lacking DNA repair domains. The hypermethylation of *HLTF* promoter was first identified in colon cancer [11] and was reported in other types of cancers, including gastric cancers [12–16]. It was shown in HeLa cells that *HLTF* mRNA was alternatively spliced in the exons 19 to 22 region, resulting in the expression of shorter truncated protein forms. The distinctive character of the *HLTF* spliced mRNA variants (I21R) is that they contain the intron 21 between exons 21 and 22. To date, the expression of HLTF protein forms was reported in head and neck, cervix and thyroid [17–20] cancers and associated with a poor prognosis [16].

In lung cancer, one study assessed the hypermethylation of *HLTF* in a cohort of 54 patients with NSCLC [21]. Promoter hypermethylation was found in 21 patients (39.6%), including 9/20 squamous cell carcinoma (SCC) and 12/33 adenocarinoma (ADC). Patients whose tumors harboured *HLTF* hypermethylation had shorter survival, in comparison with patients whose tumors had a hypomethylated *HLTF* promoter (log-rank, *p* = 0.035). So far, to our knowledge, there are no published data about the expression of HLTF (wild-type and its truncated forms) in lung cancer.

The purpose of this study is to assess the expression of wild-type (WT) and spliced variants (I21R) of *HLTF* mRNAs in NSCLC and evaluate their clinical relevance. We analyzed publicly available databases for *HLTF* in lung cancer and assessed its expression in NSCLC cell lines and in a clinically annotated cohort of 171 patients with resected stage I-II NSCLC.

## Methods

### In silico analyses

Available genomic profiling data (mutation, copy number, DNA methylation [correlation only], and mRNA expression) for *HLTF* were downloaded from cBioPortal, an online portal for accessing data from The Cancer Genome Atlas (TCGA) project and other cancer genome profiling initiatives (http://www.cbioportal.org/public-portal). Additional cancer genome profiling data were obtained from the Catalogue of Somatic Mutations in Cancer (http://cancer.sanger.ac.uk/cosmic; [22]).

To obtain gene expression estimates for individual mRNA forms, paired-end RNAseq raw read data from the TCGA project were downloaded from the Cancer Genomics Hub (https://cghub.ucsc.edu/). In brief, reads were mapped to the latest human reference assembly, hg19, using TopHat, a splice-aware short-read aligner (http://ccb.jhu.edu/software/tophat). Alignment output was then supplied to Cufflinks (http://cufflinks.cbcb.umd.edu/), which was run in the reference-guided mode to quantify the abundance of known transcripts as well as predict and estimate expression of novel isoforms. Pre- and post-alignment quality control was performed with FastQC (http://www.bioinformatics.babraham.ac.uk/projects/fastqc/) and RSeQC (http://rseqc.sourceforge.net/), respectively.

### Patient characteristics

A total of 171 patients with resected stage I-II NSCLC collected at University Health Network (Toronto, Canada) were included in this study. These patients had surgery between 1996 and 2005. The length of follow-up: median 5.4 years, range 0.1–12 years. As these patients all underwent surgery before 2005, none of them received adjuvant chemotherapy as it did only become standard after 2005. The clinical and demographic characteristics of the patient cohort are listed in Table 2.

### Cell lines and cell culture

NSCLC cell lines were purchased from the American Type Cell Collection (ATCC, http://www.atcc.org), and cultured according to ATCC recommendation. Among these, there were 33 of the adenocarcinoma (ADC) subtype (H1693, H2122, H2228, H2279, H1573, H1395, H522, H1792, H838, H1819, H4011, H2291, H2073, H1568, H920, H1993, H4006, HCC827, H3255, H23, H4019, H2126, H1437, H1944, H2009, H2405, H1373, H1355, H1975, HCC2935, A549, H650, H1650), two large cell carcinoma (H661, H4017), one mixed adenosquamous carcinoma (H647) and two of undefined histology (DFC1032, DFC1024). MGH7 cells (squamous cell carcinoma, SCC) were cultured as described [23].

### mRNA expression

Total RNA was extracted from cell lines with RNeasy Mini Kit (QIAGEN) according to the manufacturer’s instructions. RNA purity and concentration were assessed with Nanodrop (Thermo Scientific). Total RNA (input 150 ng) extracted from cell lines and patient tumours were reverse transcribed into cDNA (SuperScript III, Invitrogen). Droplet Digital polymerase chain reaction (ddPCR) was performed based on the manufacturer’s recommendations (QX200, Bio-Rad). ddPCR is a highly sensitive qPCR due to a step of sample fractioning (limiting dilutions) by generation of droplets (water:oil emulsion). It allows retrieving an absolute count of RNA copies for each sample, and is particularly indicated for low-expressed targets. Each ddPCR was performed with 22.5 ng cDNA in triplicates. Reaction conditions were as follow: ddPCR cycle was set up at 95 °C for 5 min, 40 cycles of [30 s at 95 °C and 1 min at 58 °C], 5 min at 4 °C, and finally 5 min at 90 °C. Results were analyzed with QuantaSoft (Bio-Rad), and the cut-off to define positive and negative droplets was set up at 10,000 arbitrary units of fluorescence amplitude. This signal is then used in the calculation of *HTLF* copy number by a Poisson regression (QuantaSoft, Bio-Rad).

Primers to detect either WT *HLTF* mRNA (F: 5’-GTTCAAAGATTAATGCGCT-3′ and R: 5’-AAAGACAGGAATGTTGTAAACTGAGA-3′) or *HLTF* mRNA variants I21R (F: 5’-TCCAGTTTCAAAGGTAAAGTACTC-3′ and R: 5’-GCCAGTGGTCAACAACAGAA-3′) by ddPCR were designed with Primer3 and purchased from Eurogentec. Primers were tested for nonspecific amplicons and primer dimers by visualizing PCR products on 1% agarose gels and droplet distribution profile (QuantaSoft, Bio-Rad).

### Statistical analyses

Expression levels of different variants of *HLTF* were measured in triplicates. The reliability was assessed by calculating the intra-class correlation coefficient (ICC) based on the within and between variances estimated using the variance component analysis. For the outcome analysis, the three replicates were averaged for each sample. Two outcome variables were assessed: overall survival (OS) and disease-free survival (DFS). Both were measured from surgery date. For OS, the time was calculated up to the date of death or last follow-up with death of any cause as an event; for DFS, the time was calculated up to the date of relapse, death or last follow-up with death or relapse as events. There were 71 deaths (number of events for OS) and 81 events for DFS in the cohort. The averages of the three replicates of WT and I21R *HLTF* expressions were tested for their associations with OS and DFS using the Cox proportional hazards regression. Both variants of *HLTF* were also dichotomized at their respective medians and were tested as categorical variables using the log-rank test. The percentages for OS and DFS for the high and low values of each of these covariates were calculated using the Kaplan-Meier method. A composite covariate was created by combining WT and I21R *HLTF* expression levels and a data-driven covariate was defined as “Low WT *HLTF* and High I21R *HLTF*” vs. the rest. This new covariate was also tested for its association with OS and DFS by employing the log-rank test. These covariates were tested for their association with outcome, adjusting the model for age (≤65, > 65), sex, stage (I vs. II), and histology (ADC vs. the rest) using Cox regression. All *p*-values were based on the Wald test. *HLTF* expressions (continuous) were also tested for their associations with the clinical factors (age, sex, histology, and stage) using the Mann-Whitney test. A cut-off of p ≤ 0.05 was used for statistical significance.

## Results

### In silico analysis of *HLTF* alterations in NSCLC

**Fig. 1:**
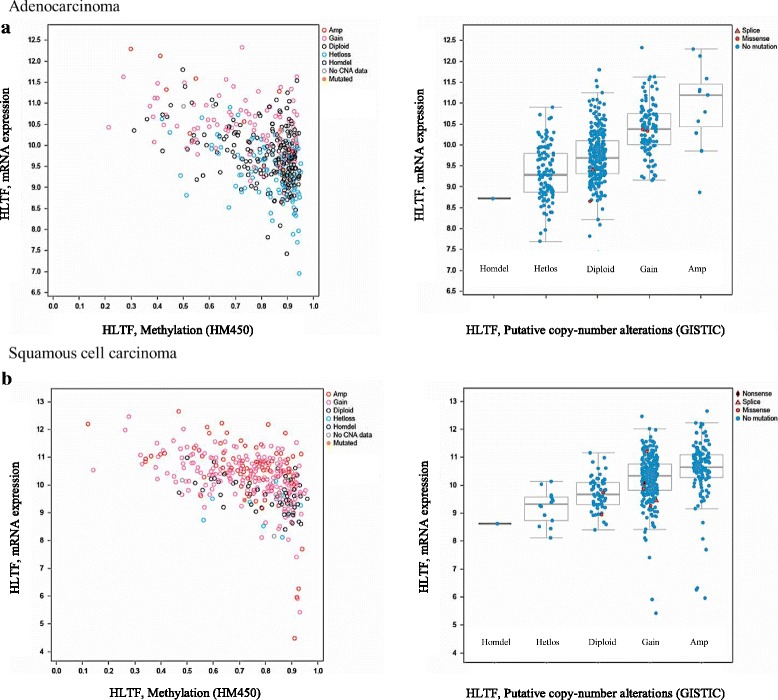
Distribution of NSCLC tumors associated with HLTF expression. **a** Adenocarcinoma. **b** Squamous cell carcinoma. HLTF expression according to HLTF methylation (left panels) and copy number alterations (CNA) (right panels)

**Fig. 2:**
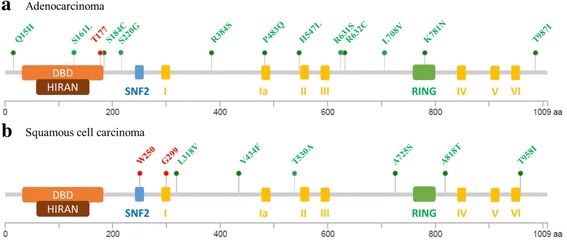
HLTF mutations in NSCLC. **a**. Lung adenocarcinoma. **b**. Lung squamous cell carcinoma. Mutation data were retrieved from TCGA and COSMIC databases. HLTF protein is depicted as a grey line with its functional domains: DNA binding domain (DBD, orange box), HIRAN (brown box), SNF2 (blue box), Helicase/ATPase I-III (yellow boxes), zinc finger RING (green box). Under the protein is a scale showing the amino-acid size. Mutation are depicted by colored dots (missense: green; spliced or stop mutation: red) with their position and the residue change

We collected available online data for *HLTF* alterations in NSCLC from TCGA (Lung ADC and SCC, TCGA Provisional 2015/02/04) and COSMIC, focusing on mutations, copy number alterations (CNAs), and methylation data. We analyzed these data in association with *HLTF* expression. The type and the frequency of the different *HLTF* alterations in ADC and SCC are reported in Table 1. When all types of molecular alteration were considered, *HLTF* was more frequently altered in SCC (438/504 cases, 83%) than ADC (266/578 cases, 46.0%; *p* < 0.0001). While a high expression of *HLTF* was more frequently reported in SCC than in ADC (28.6% vs 7.5%, *p* < 0.0001), a negative correlation between *HLTF* expression and its methylation status was found in both SCC (Pearson: − 0.475 and Spearman: − 0.484; Fig. 1) and ADC (Pearson: − 0.473 and Spearman: − 0.420; Fig. 1). *HLTF* copy number gain was more frequent in SCC (57% vs 23.3%, *p* < 0.0001) as well as high amplifications (26.1% s 2.1%, *p* < 0.0001). Conversely, loss of heterozygosity was more frequent in ADC than in SCC (22.3% vs 2.9%, respectively, *p* < 0.0001), along with diploidy (52% vs 13.5%, respectively, *p* < 0.0001). *HLTF* mutation is a rare event both in ADC and SCC, 2.2 and 1.5%, respectively (Table 1). The majority of the 13 mutations (12 missense and 1 splice site mutation) reported in ADC and 8 mutations (6 missense, 1 splice site and 1 nonsense mutations) in SCC are found in regions encoding functional domains involved in DNA binding (HIRAN domain), Sp1/Sp3 interaction (carboxyl-terminal domain), and DNA repair (SNF2_N/helicase-ATPase and RING finger domains) (Fig. 2).

**Table 1:** HLTF alterations in lung adenocarcinoma and squamous cell carcinoma

	Adenocarcinoma (*n* = 578) Number of cases, n (%)	Squamous cell carcinoma (*n* = 504) Number of cases, n (%)	*P*-value (Fisher test)
Mutation	13 (2.2%)	8 (1.5%)	0.5110
Copy number alterations			
Homozygous deletion	1 (0.1%)	1 (0.2%)	1.00
Heterozygosity loss	115 (22.3%)	15 (2.9%)	< 0.0001
Diploid	268 (52.0%)	68 (13.5%)	< 0.0001
Gain	120 (23.3%)	286 (57.0%)	< 0.0001
High level of amplification	11 (2.1%)	131 (26.1%)	< 0.0001
No data	63	3	
mRNA expression level			
High expression	43 (7.5%)	141 (28.6%)	< 0.0001
Low expression	0 (0.0%)	6 (1.2%)	0.0101
Total number of altered cases			
	266 (46.0%)	438 (83.0%)	< 0.0001

Data collected from TCGA database

### In vitro screening of *HLTF* mRNA expression in a panel of NSCLC cell lines

**Fig. 3:**
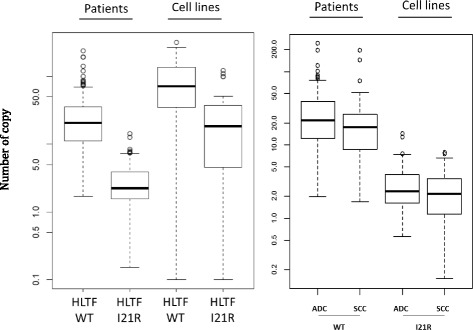
Distribution of WT and I21R HLTF expressions (number of copy) in lung adenocarcinoma (ADC) (left panel) and squamous cell carcinoma (SCC) (right panel) patients and cell lines. RNA from cell lines and tumors was extracted and reverse transcribed. A ddPCR was performed to detect WT and I21R HLTF expression by using specific primer sets. The high sensitivity of ddPCR provides an absolute count of RNA copies for each sample, displayed in Y-axis

We assessed *HLTF* mRNA expression in 39 NSCLC cell lines. Measurements for WT and I21R *HLTF* were considered reliable based on ICC values (0.878 and 0.933, respectively). Overall, the level of WT *HLTF* expression was significantly higher than the level of I21R *HLTF* expression (Fig. 3) (median 71.5 vs. 18.3 respectively, Wilcoxon signed-rank test, *p* = 4.5 × 10^− 7^).

### Assessment of *HLTF* expression in a cohort of 171 patients with NSCLC

*HLTF* expression was assessed by RT-ddPCR in 171 tumours from patients with surgically resected stage I-II NSCLC. Patient data are summarized in Table 2. Measurements for WT and I21R *HLTF* were considered reliable based on ICC values (0.984 and 0.846, respectively). As in NSCLC cell lines, the level of WT *HLTF* expression was significantly higher than the level of I21R *HLTF* expression in tumours of patients with NSCLC (Fig. 3) when considering all patients (median 20.6 vs. 2.2 respectively, Wilcoxon signed-rank test, *p* = 2.2 × 10^− 16^). There was no difference between SCC and ADC for WT and I21R *HLTF* expressions (*p* = 0.09 and 0.17, respectively). Overall, the level of *HLTF* expression was lower in tumours from patients than in cell lines (WT *HLTF*: 20.6 vs. 71.5, *p* = 9.3 × 10^− 11^ and I21R *HLTF*: 2.2 vs. 18.3, *p* = 2.8 × 10^− 12^, Wilcoxon rank-sum test; Fig. 3).

**Table 2:** Association of WT HLTF and I21R HLTF expressions with patient clinical characteristics

Clinical factor	Categories	n	Summary WT HLTF Median (range)	p-value	Summary I21R HLTF Median (range)	*p*-value
Age	Age < 65	57	20.7 (2–193)	0.63	2.1 (0.7–12.7)	0.54
	Age > =65	114	20.5 (1.7–240)		2.3 (0.2–14.3)	
Sex	F	78	20.4 (2–121)	0.84	2.4 (0.2–8.4)	0.71
	M	93	20.6 (1.7–240)		2.2 (0.6–14.3)	
Stage	I	121	19.6 (1.7–240)	0.46	2.1 (0.2–14.3)	0.91
	II	50	23.8 (3.5–195)		2.3 (0.6–7.6)	
Histology	ADC	122	21.5 (2–240)	0.061	2.3 (0.6–14.3)	0.19
	OTH	49	17.5 (1.7–195)		2.1 (0.2–8.4)	
	ADC	122	21.5 (2–240)	0.09	2.3 (0.6–14.3)	0.17
	SCC	42	17.5 (1.7–195)		2.1 (0.2–7.9)	

*ADC* Adenocarcinoma, *OTH* Other histology types, *SCC* Squamous cell carcinoma. The Mann-Whitney test was used. A cut-off of *p* ≤ 0.05 was used for statistical significance

### HLTF and clinical characteristics

WT and I21R *HLTF* expressions were first tested for their association with patient clinical characteristics (age, sex, histology, and stage). There was no association with these characteristics and *HLTF* expression (Table 2).

Second, we considered a composite covariate combining WT and I21R *HLTF* expressions dichotomized at their median levels. Four groups were built accordingly. There was no association with patient clinical characteristics (Table 3).

**Table 3:** Association of the composite covariate combining WT HLTF and I21R HLTF expression levels (n, %) with patient clinical characteristics

Clinical factor	Categories	n	WT < =20.6 Mut < =2.23	WT < =20.6 Mut > 2.23	WT > 20.6 Mut < =2.23	WT > 20.6 Mut > 2.23	*p*-value
Age	Age < 65	57	20 (32.8%)	8 (32%)	10 (45.5%)	19 (30.2%)	0.62
	Age > =65	114	41 (67.2%)	17 (68%)	12 (54.5%)	44 (69.8%)	
Sex	F	78	26 (42.6%)	13 (52%)	11 (50%)	28 (44.4%)	0.85
	M	93	35 (57.4%)	12 (48%)	11 (50%)	35 (55.6%)	
Stage	I	121	46 (75.4%)	17 (68%)	15 (68.2%)	43 (68.3%)	0.81
	II	50	15 (24.6%)	8 (32%)	7 (31.8%)	20 (31.7%)	
Histology	ADC	122	40 (65.6%)	16 (64%)	17 (77.3%)	49 (77.8%)	0.78
	OTH	49	21 (34.4%)	9 (36%)	5 (22.7%)	14 (22.2%)	
	ADC	122	20 (32.8%)	8 (32%)	10 (45.5%)	19 (30.2%)	0.35
	SCC	42	41 (67.2%)	17 (68%)	12 (54.5%)	44 (69.8%)	

*ADC* Adenocarcinoma, *OTH* Other histology types, *SCC* Squamous cell carcinoma. The Mann-Whitney test was used. A cut-off of *p* ≤ 0.05 was used for statistical significance

### HLTF expression and outcome

A univariate analysis (Cox proportional hazard regression model and log-rank test) was first performed to assess the association between WT and I21R *HLTF* expression levels, and OS and DFS. The mRNA expression measures were sequentially considered as continuous and as dichotomous variables. There was no significant association of each variable with OS and DFS (Table 4).

**Table 4:** Univariate analyses of the association of HLTF expression with overall survival and disease-free survival

Outcome			Overall survival (OS)				Disease-free survival (DFS)			
Category		n	Estimate at 5 years	Logrank *p*-value	HR (95% CI)^a^	Wald *p*-value	Estimate at 5 years	Logrank *p*-value	HR (95% CI)^a^	Wald *p*-value
WT	<=20.6	86	61%	0.63	1.03 (0.97–1.1)	0.34	48%	0.14	1.01 (0.95–1.08)	0.72
	> 20.6	85	64%				61%			
I21R	<=2.23	83	63%	0.65	1.03 (0.92–1.15)	0.61	58%	0.73	1.02 (0.93–1.13)	0.64
	> 2.23	88	61%				49%			
Composite covariable	Wt > 20.6 or Mut < =2.23	146	65%	0.47	1.26 (0.67–2.34)	0.48	59%	0.0096	1.96 (1.17–3.3)	0.011
	Wt < =20.6 and Mut > 2.23	25	50%				25%			

Cox proportional hazard regression model and log-rank test were used. A cut-off of *p* ≤ 0.05 was used for statistical significance

^a^Note: HRs for WT HLTF represent the increase of the hazard for 10 units increase in the WT HLTF

**Fig. 4:**
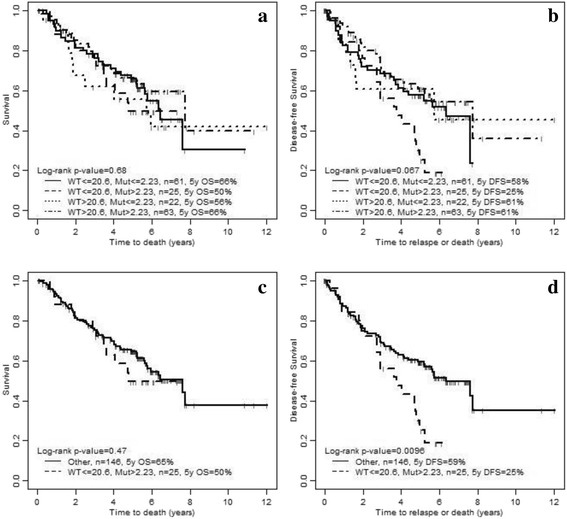
Association of HLTF expression with OS (**a**, **c**) and DFS (**b**, **d**). Four groups of patients were built, based on the combined covariates of WT and I21R HLTF expression levels. WT: wild-type HLTF. Mut: I21R HLTF. In A and C, the four groups were considered independent from each other. In C and D, the group “low WT HLTF-High I21R HLTF” was compared with the other ones, which were combined in one group called “Other”

The association of the combined covariates of WT and I21R *HLTF* expression with OS and DFS were analyzed by the log-rank test. When considering the four groups, the « Low WT *HLTF*-High I21R *HLTF* » group showed a trend for a poorer DFS, but did not reach statistical significance (DFS at 5 years = 25%, log-rank *p* = 0.067), compared with the three other groups. We thus compared this group (Low WT *HLTF*-High I21R *HLTF*) to the three other groups combined. There was no statistical difference in OS (HR 1.26, CI 0.67–2.34; *p* = 0.48), but the DFS was significantly worse in this group (HR 1.96, CI 1.17–3.30; *p* = 0.011) (Table 4 and Fig. 4d).

A multivariate analysis (Cox proportional hazard regression model) was performed to include age, sex, stage, histology, and *HLTF* expression; WT alone (model 1), I21R alone (model 2) and the 2 composite groups (model 3) were considered (Table 5). *HLTF* expression (for each model) was not associated with OS. The expressions of WT (model 1) and I21R *HLTF* (model 2) were not associated with DFS. However, the shorter DFS of the group « Low WT *HLTF*-High I21R *HLTF* » (model 3) as compared with the other groups remained significant (HR 1.98, CI 1.15–3.4, *p* = 0.014; Table 5).

**Table 5:** Multivariate analyses of the association of HLTF expression with survival and disease-free survival

Models^a^	Overall survival (OS)			Disease-free survival (DFS)		
	HR	95% CI	*p*-value	HR	95% CI	*p*-value
Model 1: adjusted effect of WT HLTF (for 10 units)	1.02	0.96–1.08	0.58	1	0.94–1.06	0.94
Model 2: adjusted effect of I21R HLTF	1.01	0.9–1.13	0.84	1	0.9–1.11	0.97
Model 3: adjusted effect of HLTF WT < =20.6 & I21R > 2.23 vs. the rest	1.21	0.64–2.28	0.56	1.98	1.15–3.4	0.014

Cox proportional hazard regression model was used. A cut-off of *p* ≤ 0.05 was used for statistical significance

^a^All models are adjusted for age, sex, stage and histology

## Discussion

The purpose of this study was to assess the expression of WT and variant forms of *HLTF* mRNAs in NSCLC and evaluate their clinical relevance. Our hypothesis was that the expression of *HLTF* mRNA variant I21R has a poor prognosis on patients with NSCLC. In head and neck, cervix and thyroid cancers, the expression of HLTF truncated protein has been associated with poor outcome [16–20]. The present study showed that in a cohort of 171 patients, the combination of low expression of WT *HLTF* transcript and high expression of I21R *HLTF* transcript was associated with poor prognosis in early stage NSCLC.

Overall, in silico analysis showed that *HLTF* alterations, including gene amplifications, high expression, and methylation occurred more frequently in SCC than in ADC. Mutations in *HLTF* were rare in both ADC and SCC; however, the mutations observed in ADC were different from those found in SCC. In ADC, mutations occur in DNA binding domain and DNA repair domains (Fig. 2), which might alter HLTF transcriptional and DNA repair abilities. Conversely, in SCC, mutations did not occur in functional domains but there are 2 nonsense mutations leading to the expression of a shorter protein containing only the DBD. This suggests that these shorter proteins would only have transcriptional activity. Further investigations are required to assess the functional consequence and potential clinical impact of these mutations in cancer. Copy number alterations were also found to be different between ADC and SCC; high amplifications were rare in ADC, but 83% of SCC have either a gain (57%) or an amplification (26%) of *HLTF*. These observations are consistent with the fact that *HLTF* is located on chromosome 3q, which is frequently amplified in SCC. We also analyzed the association of *HLTF* expression with its methylation status in both NSCLC types. In both ADC and SCC, there was a negative correlation between methylation and *HLTF* expression, but a high expression was more frequently seen in SCC, which might be related to the higher frequency of gene copy number. Intriguingly, we did not notice any difference in *HLTF* expression levels (WT and I21R) between ADC and SCC by RT-ddPCR. This discrepancy may be possibly explained by the fact that we assessed *HLTF* mRNA variants separately, while data reported in cBioportal considered only WT *HLTF* expression without distinguishing the variants.

In the available online data, only WT *HLTF* expression was assessed. To our knowledge, to date the expression of the *HLTF* spliced variants with intron 21 retention (I21R) has not been assessed. Using the RT-ddPCR with specific primers that we constructed, we were able to evaluate the expression of WT *HLTF* mRNA and its spliced variants I21R. Spliced variants I21R lead to the expression of shorter protein forms, which are thought to disturb WT HLTF function and act as oncogene proteins [16]. Studies in head and neck, cervix and thyroid cancers showed that the expression of such shorter proteins was associated with poor prognosis [17–20]. They replace WT HLTF progressively and accumulate along the carcinogenic process, most likely due to their higher stability compared with WT HLTF. It was reported that the I21R transcripts have a lower abundance than the WT *HLTF* transcript in mouse heart and brain transcriptomes [7, 24]. We analyzed RNA-seq data from TCGA for the presence of the intron 21 sequence and found that its expression was a rare event in NSCLC. In both the NSCLC cell lines and the 171 resected NSCLC from patients, WT *HLTF* levels were significantly higher than I21R *HLTF*.

Castro et al. studied the methylation for several genes including *HLTF* in NSCLC and reported that patients with *HLTF* methylation have shorter survival [21]; this study represents the only study of *HLTF* in lung cancer. They reported *HLTF* methylation frequency for NSCLC and did not observe any significant difference for *HLTF* methylation between ADC and SCC (12/33 vs. 9/20, respectively; *p* = 0.57, Fisher exact test). cBioportal does not provide gene methylation frequency but only correlations with the expression of a given gene. In both ADC and SCC, we observed a negative correlation between *HLTF* expression and methylation. Interestingly, *HLTF* expression was affected more by the variation in *HLTF* copy number than its promoter methylation status.

## Conclusion

So far to our knowledge, our study is the first to assess the clinical impact of WT and variant forms of *HLTF* expression in patients with NSCLC. TCGA in silico analysis of *HLTF* alterations including mutations, amplification, and mRNA expression modifications were more frequent in SCC than in ADC. In NSCLC cell lines and patient samples, both the expressions of WT and spliced I21R *HLTF* mRNAs were detected, but with the latter at lower levels. In a cohort of 171 patients with resected stage I-II NSCLC, the combination of a low WT *HLTF* expression with a high I21R *HLTF* expression was associated with shorter DFS both in univariate and multivariate analyses. Surgically resected early stage NSCLC are very heterogeneous and no prognostic factor has been clinically validated for the risk of relapse. Very likely, a panel of several biomarkers will be necessary to predict tumour with poor prognostic; that would therefore require more intensive follow-up and treatment. If validated in independant cohorts, the combination of low WT and high I21R *HLTF* might belong to this biomarker panel for the prognostic of surgically resected NSCLC. As detailed in a review article we published recently [16], the *HLTF* gene could be involved in various ways during the stages of tumour initiation and progression, by its ability to alternatively express proteins of different sizes with distinct functions ranging from tumour suppressor to oncoprotein. The involvement of alternative RNA splicing in producing tumour promoting proteins is a process that does not require inactivating mutation of a tumour suppressor gene and might be an underestimated carcinogenic mechanism. Further studies should precisely investigate the functions of these HLTF protein forms and their role in cancer development.

## Abbreviations

ADC: Adenocarcinoma
CNA: Copy number alteration
DFS: Disease-free survival
HLTF: Helicase-like transcription factor
HR: Hazard ratio
ICC: Intraclass coefficient
NSCLC: Non-small cell lung cancer
OS: Overall survival
RT-ddPCR: Reverse transcription-droplet digital PCR
SCC: Squamous cell carcinoma
WT: Wild-type
